# 1813. A Retrospective Review of the Safety and Efficacy of Dalbavancin at Ireland's Largest Acute Medical Hospital

**DOI:** 10.1093/ofid/ofad500.1642

**Published:** 2023-11-27

**Authors:** Peter Conlon, Ciaran Bannan, Mary Kelly, Claire Williamson

**Affiliations:** Beaumont Hospital Dublin, Dublin 12, Dublin, Ireland; Trinity School of Medicine, Dublin, Dublin, Ireland; Pharmacy Department, St James's Hospital, Dublin, Ireland, Dublin, Dublin, Ireland; Pharmacy Department, St James's Hospital, Dublin, Ireland, Dublin, Dublin, Ireland

## Abstract

**Background:**

Dalbavancin is second-generation lipoglycopeptide antibiotic. It is licensed in Ireland and the United States for the management of acute skin and soft tissue infections. International colleagues report treatment success in its use for patients who are unable to stay in the hospital for prolonged periods and not suitable for OPAT [1]. We reviewed the use of Dalbavancin at St James's Hospital Dublin, which is the largest hospital in Ireland with over 700 acute beds. We are located in Dublin’s inner city which experiences significant socio-economic deprivation.

**Methods:**

We used the electronic patient records at St James's Hospital to capture all uses of Dalbavancin from August 2018 to September 2022.

We established baseline demographics, indication, microbiological characteristics if there was oversight by ID/micro, safety, and treatment outcomes.

**Results:**

< We identified 23 patients who met the inclusion criteria

Base demographic:

The most common indication was endovascular infection (35%) and skin and soft tissue infection (35%). (Figure 1)

Most patients were not bacteremic. Staph aureus was the most common organism identified on blood culture (figure 2)

322 hospital bed were saved which equates to 257,600 euro

0 patients had an adverse drug reaction

100% of cases were discussed with micro or ID

**Conclusion:**

Dalbavancin is a safe and effective drug. It is another tool that can be used when caring for patients who are experiencing unstable housing and challenging social circumstances. Our experience has found 0 adverse events, 69% of patients experienced treatment success (this increases to 88.8% if we exclude those lost to follow up). This is an important finding considering on 17% of indications were considered ‘on label’.

Dalbavancin can save money on hospital beds and also reduces non-recyclable plastic associated with repeated doses of IV antibiotics.

**Disclosures:**

**All Authors**: No reported disclosures

**Table:** 

Male	19 (82.6%)
Average age	42
On label (SSTI and non bacteremic)	4 (17.3%)
Unstable housing	11 (47.8%)
PWID	18 (78.3%)
PLWHIV	3 (13%)
Followed up	18 (78.3%)
Bacteraemic	9 (39%)
Adverse drug reaction	0 (0%)
Treatment success	16 (69.5%)

